# Spinal anesthesia is a grossly underutilized gold standard in primary total joint arthroplasty: propensity-matched analysis of a national surgical quality database

**DOI:** 10.1186/s42836-023-00163-w

**Published:** 2023-02-09

**Authors:** Andre C. Ferreira, Chun Wai Hung, Ramesh B. Ghanta, Melvyn A. Harrington, Mohamad J. Halawi

**Affiliations:** 1 Baylor College of Medicine, Houston, TX 77030 USA; 2 Department of Orthopedic Surgery, Baylor College of Medicine, 7200 Cambridge Street, Suite 10A, Houston, TX 77030 USA

**Keywords:** Anesthesia, Arthroplasty, Outcomes, Utilization, Value care

## Abstract

**Background:**

There is currently no consensus regarding the optimal anesthetic technique for total hip and knee arthroplasty (THA, TKA). This study aimed to compare the utilization rates and safety of spinal *vs*. general anesthesia in contemporary THA/TKA practice.

**Methods:**

Using the American College of Surgeons National Surgical Quality Improvement Program (ACS-NSQIP), a retrospective review of 307,076 patients undergoing total hip or knee arthroplasty under either spinal or general anesthesia between January 2015 and December 2018 was performed. Propensity matching was used to compare differences in operative times, hospital length of stay, discharge destination, and 30-day adverse events. The annual utilization rates for both techniques between 2011 and 2018 were also assessed.

**Results:**

Patients receiving spinal anesthesia had a shorter length of stay (*P* < 0.001) for TKA while no statistical differences in length of stay were observed for THA. Patients were also less likely to experience any 30-day complication (OR = 0.82, *P* <0.001 and OR = 0.92, *P* < 0.001 for THA and TKA, respectively) while being more likely to be discharged to home (OR = 1.46, *P* < 0.001 and OR = 1.44, *P* < 0.001 for THA and TKA, respectively). Between 2011 and 2018, spinal anesthesia utilization only increased by 1.4% for THA (*P* < 0.001) and decreased by 0.2% for TKA (*P* < 0.001), reaching 38.1% and 40.3%, respectively.

**Conclusion:**

Spinal anesthesia remains a grossly underutilized tool despite providing better perioperative outcomes compared to general anesthesia. As orthopedic surgeons navigate the challenges of value-based care, spinal anesthesia represents an invaluable tool that should be considered the gold standard in elective, primary total hip and knee arthroplasty.

## Introduction

There is no current consensus in the arthroplasty community on what anesthetic technique is the gold standard for primary total hip arthroplasty (THA) and total knee arthroplasty (TKA). Spinal anesthesia (SA) and general anesthesia (GA) are the most common anesthesia types. Although there are some absolute contraindictions to SA (patient refusal, spinal pathology, coagulopathy, *etc.*), much of the choice between GA and SA often comes down to provider preference in the absence of contraindications [1]. While some studies have demonstrated decreased postoperative complications using SA [2–4], others have shown it to be non-superior to modern GA techniques even for rapid recovery [5–7]. Furthermore, most previous reports were derived from single institutions, included small sample sizes, or predated modern-day enhanced recovery protocols [8–10]. As arthroplasty surgeons adapt to value-based payment programs and the challenges of COVID-19, revisiting the role of anesthesia warrants further investigation.

Healthcare reform, expansion of bundled payment programs and the dramatic disruptions in electively scheduled THA and TKA caused by the novel coronavirus disease of 2019 (COVID-19) have triggered accelerated transition to perform those procedures on an outpatient basis, thereby freeing up hospital beds for COVID-19 patients [11, 12]. Among the most widely emphasized pillars for the success of this paradigm shift are preoperative optimization of medical comorbidities, patient preparation, multimodal analgesia, blood management, and discharge to home [13]. A less commonly discussed yet important intervention, especially in the context of the COVID-19 pandemic, is the role of anesthesia. Compared to general endotracheal intubation, SA can conserve vital resources including ventilators and oxygen while minimizing the costs of care, risk of COVID-19 transmission, and the potential side effects associated with GA.

This retrospective cohort study utilized a national quality improvement database to compare the utilization rates and safety of SA *vs.* GA in contemporary THA and TKA practices. We hypothesized that SA would provide superior postoperative outcomes. The results of this study would help guide best practice recommendations for arthroplasty care in a rapidly evolving healthcare environment.

## Materials and methods

### Patient selection and allocation

This study was exempt from institutional review board review because it utilized a de-identified patient database. A retrospective review of The American College of Surgeons National Surgical Quality Improvement Program (ACS-NSQIP) database was conducted. Between January 2015 and December 2018, patients undergoing procedures with the Current Procedural Terminology (CPT) codes 27,130 (arthroplasty, acetabular, and proximal femoral prosthetic replacement with or without autograft or allograft) and 27,447 (arthroplasty, knee, condyle, and plateau, medial and lateral compartment, with or without patellar resurfacing) were identified. Patients with emergent, non-elective, and revision, and bilateral procedures were excluded. Patients who did not undergo either general or spinal anesthesia were also excluded.

Demographic, comorbid, and perioperative factors were collected. Demographic variables included age, sex, race/ethnicity, and body mass index (BMI). Comorbidities included tobacco use within 1 year before surgery, chronic steroid use, diabetes mellitus, hypertension, chronic obstructive pulmonary disease, congestive heart failure, bleeding disorders, history of metastatic cancer, anemia, dyspnea, and chronic kidney disease. Perioperative variables included the American Society of Anesthesiologists (ASA) physical classification, surgical indication (primary *vs*. non-primary osteoarthritis), and operative time (min).

### Data assessment

Patients were divided into SA and GA groups. We assessed hospital length of stay (LOS), discharge destination, and 30-day postoperative adverse events. Adverse events included medical complications (*C. difficile* infection, septic shock/sepsis, myocardial infarction/cardiac arrest, stroke/cerebral vascular accident, urinary tract infection, acute renal failure, renal insufficiency, pneumonia), surgical complications (surgical site infection, deep vein thrombosis, ventilation longer than 48 h, pulmonary embolism, unplanned intubation), readmissions, reoperations, or mortality.

### Statistical analysis

Propensity matching was used to control for potential selection biases in treatment based on patient characteristics and demographics. Patients in the two cohorts were matched one-to-one with replacement via Nearest Neighbor Matching logistic regression. A caliper width of 0.2 was used to match patients to the nearest propensity score. Matching criteria included all demographic factors, comorbidities, procedure (THA or TKA), and year of operation. The only criterion excluded from matching was race/ethnicity. After matching, 206,608 out of 307,076 patients remained. Each cohort contained 103,304 patients matched to each other one-to-one. Power analysis was conducted via G*Power 3.1 on expected total complications, using the results of a previous study by Warren *et al.* [14]. Logistic regression testing with an expected R-squared of 0.81 yielded a minimum total sample size of 17,102 to reach a power of 0.80. With a final post-match sample size of 206,608, our study was sufficiently powered for analysis.

Continuous variables (*e.g.*, age, BMI) were reported as mean and standard deviation. All continuous variables of this study, including age, BMI, and ASA classification, conformed to the normal distribution and Welch’s *t*-test was used to compare cohorts before and after propensity matching. Categorical variables were reported as absolute frequencies and percentages and compared using Pearson’s Chi-squared test before and after propensity matching. All reported *P* values were two-sided and a *P* value of less than 0.05 was considered significant.

Multivariate logistic regression was performed for binary primary outcomes while controlling for significant demographics, comorbidities, and perioperative variables. Multivariate linear regression was used for the length of stay and operation time. Results for such analysis were presented in odds ratios (OR) and 95% confidence intervals (CI). Mixed effect logistic regression comparing postoperative outcomes between the study groups was performed separately for THA and TKA. The annual trends for anesthesia modality utilization from 2011 to 2018 were also analyzed using a univariate mixed-effect logistic regression. All data analyses were performed using R studio 2021 (RStudio PBC, Boston, MA, USA).

## Results

Both study cohorts were successfully matched in terms of demographic, comorbid, and perioperative factors (*P* > 0.05 for all variables, Table 1). THA patients who received spinal anesthesia experienced lower rates of 30-day complications (OR = 0.82, *P* < 0.001), readmissions (OR = 0.85, *P* < 0.001), and reoperations (OR = 0.89, *P* < 0.001) compared to patients who received GA. Similarly, the SA cohort was more likely to be discharged to home (OR = 1.46, *P* < 0.001). There were no significant differences in 30-day mortality rates (Table 2).

**Table 1:** Baseline characteristics of the study cohorts

	General anesthesia	Spinal anesthesia	***P*** Value
*n* (% of total)	103,304 (50.0%)	103,304 (50.0%)	—
**Demographic Characteristics**			
Age (years)	67.1 ± 9.8	67.1 ± 9.7	0.451
Sex (male)	40,744 (39.4%)	41,436 (40.1%)	0.173
BMI	31.7 ± 6.5	31.7 ± 6.5	0.659
**Comorbidities**			
Tobacco smoking within 1 year	8,344 (8.1 %)	8,574 (8.3%)	0.067
Chronic steroid use	3,113 (3.0%)	3,118 (3.0%)	0.959
Diabetes	14,958 (17.3%)	14,952 (14.5%)	0.975
Hypertension	59,800 (57.9%)	59,601 (57.7%)	0.369
COPD	3,373 (3.3%)	3,353 (3.2%)	0.814
Congestive heart failure	247 (0.2%)	232 (0.2%)	0.522
Bleeding Disorders	1,303 (1.3%)	1,290 (1.2%)	0.813
History of metastatic cancer	120 (0.1%)	115 (0.1%)	0.794
Anemia	26,240 (25.4%)	26,475 (25.6%)	0.236
Dyspnea	4,801 (4.7%)	4,841 (4.7%)	0.684
Chronic kidney disease	2,496 (2.4%)	2,551 (2.5%)	0.441
**Perioperative Characteristics**			
Primary osteoarthritis	100,682 (97.5%)	100,682 (97.5%)	> 0.999
Total Hip Arthroplasty	35,022 (33.9%)	35,253 (34.1%)	—
Total Knee Arthroplasty	68,282 (66.1%)	68,051 (65.9%)	—
ASA Physical Classification	2.4 ± 0.6	2.4 ± 0.6	0.675

*ASA* American Society of Anesthesiologists, *CHF* congestive heart failure, *CKD* chronic kidney disease, *COPD* chronic obstructive pulmonary disease

Continuous variables were compared between general anesthesia patients and spinal anesthesia patients using Welch’s *t*-test with two-tailed *P*-values with general anesthesia patients as the control group. Categorical variables were compared between general anesthesia patients and spinal anesthesia patients using Pearson’s Chi square test with general anesthesia patients as the control group

**Table 2:** Results of multivariate logistic regression analyses comparing outcomes of spinal to general anesthesia (control)

Outcome	Total hip arthroplasty		Total knee arthroplasty	
	Propensity-adjusted Odds ratio (95% CI)	***P***-Value	Propensity-adjusted Odds ratio (95% CI)	***P***-Value
Discharge to Home	1.46 (1.39 – 1.52)	< 0.001	1.44 (1.40 – 1.48)	< 0.001
Any 30-day Complications	0.82 (0.75 – 0.86)	< 0.001	0.92 (0.88 – 0.96)	< 0.001
Surgical Complications	0.84 (0.75 – 0.95)	0.004	0.91 (0.85 – 0.98)	0.014
Medical Complications	0.83 (0.73 – 0.94)	< 0.001	0.88 (0.80 – 0.96)	0.005
Readmission	0.85 (0.79 – 0.92)	< 0.001	0.91 (0.86 – 0.96)	0.001
Reoperation	0.89 (0.73 – 0.90)	< 0.001	0.89 (0.81 – 0.98)	0.015
Mortality	0.85 (0.52 – 1.40)	0.527	1.03 (0.75 – 1.41)	0.868
Operative time (minutes)	-12.09 ± 0.19^a^	< 0.001	-8.02 ± 0.20^a^	< 0.001
Length of Stay (days)	0.00 ± 0.02^a^	0.820	-0.09 ± 0.01^a^	< 0.001

^a^Operative time and LOS are reported as unstandardized beta coefficients representing change in unit time if spinal anesthesia was used

For TKA, patients who received SA were less likely to have 30-day complications (OR = 0.92, *P* < 0.001), readmissions (OR = 0.91, *P* = 0.001), and reoperations (OR=0.89, *P* = 0.015) compared to the GA cohort. Similarly, the SA cohort was more likely to be discharged home (OR = 1.44, *P* < 0.001). There were no significant differences in 30-day mortality rates (Table 2).

Spinal anesthesia was associated with a mean reduced operative time of 12.09 minutes (*P* < 0.001) and 8.02 min (*P* < 0.001) for THA and TKA, respectively. Additionally, SA was associated with a similar reduction in overall LOS in TKA by 0.09 days or 2.16 h (*P* < 0.001); however, no statistical difference in overall LOS was observed between SA and GA in THA (*P* = 0.820). The differences in operative time and LOS are summarized at the bottom of Table 2.

Table 3 demonstrates the trends in the utilization of SA within our study cohort from 2011 to 2018. For THA, SA utilization only increased by 1.4% (*P* < 0.001), reaching 38.1% in 2018. For TKA, SA utilization decreased by 0.2% (*P* < 0.001), reaching 40.3% in 2018.

**Table 3:** Utilization rates of spinal anesthesia between 2011 and 2018

Procedure	2011	2012	2013	2014	2015	2016	2017	2018	***P*** Value
THA	36.7%	37.2%	35.2%	35.1%	36.5%	37.5%	36.2%	38.1%	< 0.001
TKA	40.5%	37.0%	35.5%	37.9%	39.6%	40.1%	40.2%	40.3%	< 0.001

*THA* total hip arthroplasty, *TKA* total knee arthroplasty

## Discussion

In this study, we asked two simple questions: (1) Does SA provide superior benefits compared to modern GA techniques? (2) What are the current utilization rates of SA in primary, unilateral THA/TKA? Consistent with our hypothesis, we found that the SA group had significantly shorter operative time and LOS while also lower rates of non-home discharge, 30-day complications, readmissions, and reoperations for both THA and TKA compared to the GA group. Despite the above findings, the national utilization rates of SA have increased by only 1.4% for THA while decreasing by 0.2% for TKA in the past decade, with overall utilization remaining under 50%.

Our findings on the advantages of SA are consistent with a few previous reports. In a retrospective review of the ACS-NSQIP database from 2011 to 2016, Warren *et al.* [14] found that SA was associated with lower rates of 30-day complications for total joint arthroplasty (TJA) compared to GA. In another study, Basques *et al*. found that GA was associated with increased adverse events, including prolonged postoperative ventilator use, unplanned reintubation, stroke, cardiac arrest, blood transfusion and even longer operative time, but this study was limited by an earlier time period from 2010–2012 [15]. In our study, we used a more contemporary ACS-NSQIP sample reflecting modern-day enhanced perioperative pathways (multimodal analgesia, intraoperative tranexamic acid use, *etc.)* that were not routinely used between 2010 and 2016. This is important because our findings indicate that SA remains a relevant and equally important intervention even when considering other improvements to recovery protocols. In another retrospective review of 3018 THA and 5389 TKA patients from a single institution, Luzzi *et al.* [16] demonstrated a lower 90-day complication rate in those who received SA compared to GA. Similarly, Kendall *et al*. compared GA *vs.* SA for TKAs, and found that higher minor adverse events were associated with GA, although this study was limited to only outpatient TKAs [17]. The reduction of postoperative complications as demonstrated in our study and others is especially relevant as bundled payment programs become more mainstream. Such events are often termed "bundle busters" because they are clinically as well as financially detrimental to institutions [16]. In this regard, SA represents a modifiable factor to help mitigate the risk of postoperative complications.

Another advantage of SA in a value-based era, as demonstrated in this study, is its impact on reducing LOS and non-home discharge, representing two areas where SA confers massive cost savings to the healthcare system. LOS is often used as a surrogate for resource utilization and cost drivers [18]. In a national cost analysis study of Medicare patients undergoing TJA, Sibia *et al.* [19] found that a reduction of LOS by one day resulted in savings of over $2000 for THA and $1800 for TKA per episode of care. With respect to discharge destination, Nichols *et al.* [20] showed, in a retrospective review of healthcare claims from the Truven MarketScan Database, that home discharge was associated with mean savings greater than $3100 per TKA patient and $4400 per THA patient compared to discharge to skilled nursing facilities. Given how enormous these cost savings are when generalized to an aging population in which TJA volume is expected to sharply increase over the next few decades, spinal anesthesia can play a critical role in optimizing outcomes while lowering the costs of care.

Even with previous reports on the benefits of spinal anesthesia, our utilization rate data illustrated that the technique remains grossly underutilized. Perhaps, this finding is attributed to a lack of consensus regarding the optimal anesthetic technique for TJA. In a retrospective review of 1527 primary TJAs at a single institution, Stambough *et al.* [5] reported that modern GA techniques were no inferior to SA in terms of LOS, physical therapy participation on the day of surgery, readmissions, and reoperations. In another recent trial that randomized 120 THA patients to either GA or SA, Harsten *et al.* [21] found that GA actually produced a shorter LOS and decreased nausea and orthostatic hypotension. Spinal anesthesia is also associated with its unique own set of complications which are not familiar to orthopedic surgeons which can lead to a decreased utilization. These include a perceived possibility of a failed spinal anesthesia to generate optimal surgical conditions (often cited to be about 10%), hypotension, prolonged motor weakness, postoperative urinary retention, post-dural puncture headaches, cauda equina syndrome, and transient neurological syndrome [22].

To our knowledge, no prior studies have examined the temporal utilization trends of spinal *vs*. general anesthesia in THA/TKA. While GA is the tried-and-true method of achieving sedation and analgesia, SA for THA/TKA is still a relatively new technique. As such, we conjecture that many surgeons and institutions may be slower adoptees of relatively "newer techniques". We believe that, as publications espousing the benefits of spinal over general anesthesia continue to emerge, increased adoption of spinal anesthesia will occur. In fact, the American Academy of Orthopedic Surgeons clinical practice guidelines recommend the use of SA over GA for THA and TKA [23, 24]. Therefore, there is no evidence-based reason for the utilization rates of SA to be low. One of the main purposes of this paper was to bring the relatively static utilization rates to the attention of both our orthopedic and anesthesia colleagues , and to promote increased utilization of SA given the benefits to our patients.

The unprecedented COVID-19 pandemic has affected orthopedic surgery particularly harshly. One of the unique challenges faced by the medical community has been the need to conserve precious healthcare resources [25]. Spinal anesthesia represents a perfect opportunity to do so. Compared to GA, SA eliminates the need for ventilators, allows for quicker recovery postoperatively, facilitates performing surgery in outpatient or ambulatory settings, and maximizes the rate of discharge to the home. In an environment where patients aim to avoid contact with healthcare facilities, the opportunity to discharge patients home rather than too crowded rehabilitation or nursing home facilities is ideal. Apart from eliminating dependence on ventilators, SA also minimizes the risk of viral transmission in the operating room that occur with endotracheal intubation for general anesthesia. For all of these reasons, spinal anesthesia offers orthopedic surgeons the opportunity to serve as stewards of important life-saving resources that can be reserved for those in greater need, thereby moving closer to being "pandemic-proof" should we experience additional waves of viral spread threatening shutdowns of elective orthopedic operations.

This study has some limitations. First, it is a retrospective review of a large database that could be subject to data entry errors. However, the ACS-NSQIP is a validated, chart abstraction-based database that is widely used in the orthopedic literature. A second limitation is that the outcomes are limited to the first 30 postoperative days, which could underestimate the true rates of adverse events. However, the majority of complications attributable to anesthetic technique tend to occur in the first 30 days after surgery [26]. Third, it is unclear what the selection criteria for SA *vs.* GA were due to the retrospective nature of this database. Lastly, with any retrospective observational data, there is always a potential for selection biases between treatment cohorts. Propensity matching was used to correct for such biases, but there might be variables outside of the dataset that influenced the recorded responses.

## Conclusion

In conclusion, SA imparts several important benefits, including shorter operative time, reduced LOS, greater home discharge, and lower rates of 30-day postoperative adverse events. These findings provide further support that SA should be considered as the "gold standard" for primary elective total hip and knee arthroplasty. As orthopedic surgeons are challenged to deliver superior outcomes at lower costs, it is important that we fully utilize all available resources including the use of spinal anesthesia. Furthermore, in an era where COVID-19 is likely to continue dominating the healthcare field for years to come, increased use of SA is one way of demonstrating stewardship in conserving healthcare resources.

## Data Availability

The datasets used and/or analyzed during the current study are available from the corresponding author on reasonable request.
